# The Global Burden of Journal Peer Review in the Biomedical Literature: Strong Imbalance in the Collective Enterprise

**DOI:** 10.1371/journal.pone.0166387

**Published:** 2016-11-10

**Authors:** Michail Kovanis, Raphaël Porcher, Philippe Ravaud, Ludovic Trinquart

**Affiliations:** 1 INSERM U1153, Paris, France; 2 Université Paris Descartes–Sorbonne Paris cité, Paris, France; 3 Assistance Publique-Hôpitaux de Paris, Hôpital Hôtel-Dieu, Centre d’Epidémiologie Clinique, Paris, France; 4 Cochrane France, Paris, France; 5 Department of Epidemiology, Columbia University Mailman School of Public Health, New York, New York, United States of America; GERMANY

## Abstract

The growth in scientific production may threaten the capacity for the scientific community to handle the ever-increasing demand for peer review of scientific publications. There is little evidence regarding the sustainability of the peer-review system and how the scientific community copes with the burden it poses. We used mathematical modeling to estimate the overall quantitative annual demand for peer review and the supply in biomedical research. The modeling was informed by empirical data from various sources in the biomedical domain, including all articles indexed at MEDLINE. We found that for 2015, across a range of scenarios, the supply exceeded by 15% to 249% the demand for reviewers and reviews. However, 20% of the researchers performed 69% to 94% of the reviews. Among researchers actually contributing to peer review, 70% dedicated 1% or less of their research work-time to peer review while 5% dedicated 13% or more of it. An estimated 63.4 million hours were devoted to peer review in 2015, among which 18.9 million hours were provided by the top 5% contributing reviewers. Our results support that the system is sustainable in terms of volume but emphasizes a considerable imbalance in the distribution of the peer-review effort across the scientific community. Finally, various individual interactions between authors, editors and reviewers may reduce to some extent the number of reviewers who are available to editors at any point.

## Introduction

The peer-review process of scientific publications became uncomfortable in the scientific community long ago [1, 2]. More recently, several voices have raised concerns about the sustainability of peer review [3–5]. In fact, the number of scientific journals and published articles has increased consistently by about 3% to 3.5% each year; in 2014 alone, about 28,100 peer-reviewed English-language journals published about 2.5 million articles [6]. In the biomedical field, MEDLINE indexed 1.1 million references from more than 5,000 journals in 2015, as compared to about 400,000 and 637,000 references in 1995 and 2005, respectively. Open access and other online journals are a factor in this growth [7].

If articles undergo peer review, the growth in scientific production inevitably puts an increasing burden on the scientific community itself to address the demand for peer review. The process frequently requires second rounds of reviews for a given submission and additional reviews when a manuscript is resubmitted after being rejected. Reviewers typically spend 4 to 5 hours reviewing a paper [8, 9]. The yearly expenditure of peer review is about 2.7 billion US dollars globally [10, 11]. This volume issue may overburden the ability of the scientific community to cope with peer-review duties [5, 12]. However, to our knowledge, we lack concrete evidence about the global demand for reviewers and whether the community self-regulates to cover the demand.

Here we assessed the sustainability of the peer-review system of the scientific publication system in the biomedical domain and how the scientific community is actually coping with the volume of submitted manuscripts.

## Methods

### Methods summary

We used a mathematical modeling approach, informed by empirical data in the biomedical domain, to compare the quantitative peer-review demand and supply.

**Fig 1:**
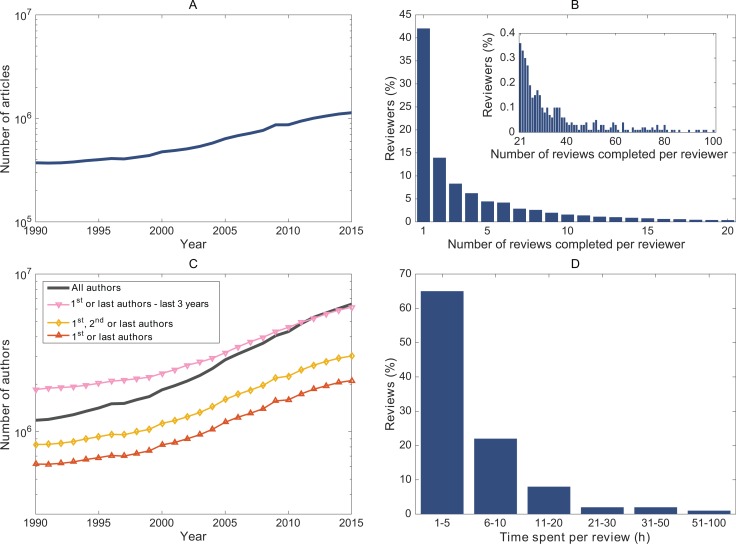
Input distributions and results derived from MEDLINE for peer review in the biomedical domain. (A) Amount of annual publications indexed by MEDLINE and the demand for reviews they generate; (B) Peer-review effort for 2015 provided by Publons. The inset shows the distribution for more than 20 reviews completed per year. Data refer to all scientific domains; (C) Number of authors who published during a given year. Data are from analyzing all annual publications indexed by MEDLINE; (D) Distribution of time spent per review. Data are from Mulligan et al. (2011) and refers to the medical domain.

We estimated the annual demand as the number of reviews and reviewers required to produce the observed annual number of published articles. The numbers of published articles were derived from MEDLINE for 1990 to 2015 (Fig 1A). We then estimated the corresponding total number of submissions. In fact, an article may be resubmitted multiple times, thus requiring additional reviews. We used the empirical distribution of the number of times papers are resubmitted from data for the biomedical domain in the 2009 Peer Review Survey, an international survey of 4,037 researchers [8]. Moreover, we assumed that 20% of submissions ultimately remained unpublished. We then estimated the corresponding total number of peer reviews (demand for reviews). Some submissions do not require any review, if they are “desk-rejected” after in-house editorial screening. We assumed that the average proportion of desk-rejected papers was 25%. Otherwise, we considered an average of 2.5 reviewers per peer-review round and that 90% of the peer-reviewed submissions went through a second round of peer review [11]. Finally, we estimated the total number of reviewers (demand for reviewers) by using the empirical distribution of individual contributions to the peer review effort (ie, the proportion of reviewers who reviewed 1, 2, 3 etc. papers in a given year) from data for 2015 in the Publons reviewer recognition platform (Fig 1B).

We estimated the annual peer-review supply as the number of potential reviewers and the number of reviews they could perform. Considering that editors typically invite past authors to be peer reviewers, we assumed that potential reviewers in a given year were researchers who co-authored at least one paper that year (Scenario 1). We also considered more stringent scenarios (in terms of co-author consideration to be a potential reviewer) in which candidate reviewers were the first or last authors of any article during the previous 3 years (Scenario 2); the first, second or last authors for the same year (Scenario 3); and the first or last authors for the same year (Scenario 4). For Scenario 2, we arbitrarily chose a time window of 3 years, which however may reflect changes in the databases that editors use to find reviewers. For each scenario, we estimated the number of potential reviewers (supply for reviewers) by counting the unique author occurrences each year from all journal articles indexed in MEDLINE from 1990 to 2015 (Fig 1C). Finally, we estimated the total number of reviews they could perform (supply for reviews) by using the empirical distribution of individual contributions to the peer-review effort.

We estimated the distribution of the proportion of research work-time devoted to peer review. For each researcher, we estimated the total time spent on peer review by using the empirical distribution of the time taken to perform each review from data for the biomedical domain in the 2009 Peer Review Survey (Fig 1D) [8].

### Estimation of demand and supply for peer-review

Let us consider *N*_*p*_ the number of articles accepted for publication. Let *N*_*u*_ be the number of articles submitted for publication but that ultimately remain unpublished. We accounted for multiple submissions after rejections, which all occurred within a given year. We assumed that both published and unpublished papers followed the same distribution of resubmissions. Let us define *R*_*i*_′, the proportion of manuscripts submitted exactly *i* times. The proportion of manuscripts submitted at least i times is $R_{i}=\sum_{k\geq i}R_{k-1}^{\prime }$. Then the total number of submissions is:

$$N_{s}=(N_{p}+N_{u})\times \sum_{i=1}^{I}R_{i}\times i$$ (1)

For simplicity, we set a maximum amount of resubmissions (*I*). For example, if 5% of papers are submitted once, 10% are submitted twice and 85% are submitted three times, then $R_{1}^{\prime }=0.05$, $R_{2}^{\prime }=0.10$, $R_{3}^{\prime }=0.85$, *R*_1_ = 1, *R*_2_ = 0.95, and *R*_3_ = 0.85. Then, $\sum_{i=1}^{3}R_{i}\times i=1\times 1+0.95\times 2+0.85\times 3=5.45$. If we further assume that 800 manuscripts were ultimately published and 200 ultimately unpublished, the total number of submissions is *N*_*s*_ = 800 × (1 + 0.95 × 2 + 0.85 × 3) + 200 × (1 + 0.95 × 2 + 0.85 × 3) = 1,000 × 5.45 = 5,450 *submissions*.

The distribution of resubmissions of published and unpublished papers might differ, but we can transform it to be the same:

$${N_{u}}^{0}\times \sum_{i=1}^{I}R_{i}^{0}\times i={N_{u}}^{0}\times \alpha \times \sum_{i=1}^{I}R_{i}\times i=N_{u}\times \sum_{i=1}^{I}R_{i}\times i$$ (2)

where *α* is a constant, ${N_{u}}^{0}=\frac{N_{u}}{\alpha }$ the real amount of unpublished papers and $R_{i}^{0}$ the real proportion of papers (re)submitted *i* times but never published. For example, if $R_{1}^{0}=1$, $R_{2}^{0}=0.85$, and $R_{3}^{0}=0.55$, then $\sum_{i=1}^{3}R_{i}^{0}\times i=4.35$. If N_u_^0^ = 100, then the total number of submissions which did not result in a publication is 370. In reality we do not know both $\sum_{i=1}^{I}R_{i}^{0}\times i$ and N_u_^0^ and it would be impossible to obtain reliable data for them. However, we know $\sum_{i=1}^{I}R_{i}\times i$ and we can represent $\sum_{i=1}^{I}R_{i}^{0}\times i$ in terms of it using a constant α. Then, we can group α and N_u_^0^ into a single constant N_u_ and work with Eq 1.

We estimated the annual demand for reviews N_reviews_ as:

$$N_{reviews}=(1-d){\times r}_{s}\times (N_{s}+\sum_{i=1}^{I}S_{i})$$ (3)

where *d* is the proportion of desk-rejected submissions, *r*_*s*_ the number of reviewers per peer review round and *S*_*i*_ the amount of papers that went to a second round of peer review in their *i*^*th*^ (re)submission. We defined *S*_*i*_ as follows:

$$S_{i}=\beta \times (N_{p}+N_{u})\times R_{i}$$ (4)

where *β* is the probability of a second peer-review round per submission that is not desk-rejected.

We can estimate *N*_*reviews*_ using a different formula, which this time involves the annual demand for reviewers *N*_*reviewers*_.

$$N_{reviews}=N_{reviewers}\times \sum_{j=1}^{J}P_{j}\times j$$ (5)

where *J* is the maximum amount of annual reviews that any reviewer performed, *j* the amount of reviews completed from a reviewer in a given year and *P*_*j*_ the proportion of reviewers who completed *j* reviews. For example, if 1,000 scientists reviewed at least one paper inside a year, 60% of them performed 1 and 40% of them 2 reviews, then N_reviews_ = 1000 × (0.6 × 1 + 0.4 × 2) = 1,400 *reviews*. Since we have two formulas estimating *N*_*reviews*_, we can estimate the annual demand for reviewers from their combination:

$$N_{reviewers}=\frac{N_{reviews}}{\sum_{j=1}^{J}P_{j}\times j}=\frac{\left(1-d){\times r}_{s}\times (N_{s}+\sum_{i=1}^{I}S_{i}\right)}{\sum_{j=1}^{J}P_{j}\times j}$$ (6)

We defined each researcher’s total amount of time available for research as follows:

$$t_{res}=work\;time\times (year-weekends-holidays)$$ (7)

### Collection and analysis of data

All data and results can be found in the accompanying Excel file (http://www.clinicalepidemio.fr/peerreview_burden/). We programmed our simulations by using MATLAB (MATLAB and Statistics Toolbox Release 2014b, The MathWorks, Inc., Natick, MA, USA). The code is available at https://github.com/kovanostra/global-burden-of-peer-review.

We used data pertaining to the biomedical domain, except to estimate *r*_*s*_ and the distribution of peer-review effort ($\sum_{j=1}^{J}P_{j}$), for which we used data pertaining to all scientific disciplines. We extracted all records indexed as “journal articles” by MEDLINE from January 1, 1990 to December 31, 2015. We downloaded the xml files for each year separately and parsed them by using a script written in Python (also available on github). We excluded all records with no author name (*e*.*g*., less than 0.001% of all articles for 2015) and indexed all authors based on their “LastName”, “ForeName” and “Initials”. We counted all the unique occurrences of authors by taking into account all these three pieces of information. For missing “ForeName” and/or “Initials”, we used only the available fields. We did not use any methods for author name disambiguation for researchers indexed under the same “LastName”, “ForeName” and “Initials”.[13, 14] We set *N*_*s*_ to be equal to the number of publications for which we identified at least one author.

We assumed that potential reviewers in a given year were researchers who co-authored at least one paper that year (Scenario 1). Then we defined more stringent scenarios (in terms of which co-authors are potential reviewers) whereby candidate reviewers were the first or last authors of any article during the previous 3 years (Scenario 2); the first, second or last authors for the same year (Scenario 3); and the first or last authors for the same year (Scenario 4). For Scenario 2, we arbitrarily chose a time window of 3 years, which however may reflect changes in the databases that editors use to find reviewers. For each scenario, we repeated the same procedure of identifying the unique occurrences of authors as described above. For each scenario, the number of authors obtained was considered to represent the potential supply of reviewers (*N*_*reviewers*–*supply*_) in any given year. We did not account for individual interactions between authors, editors and reviewers which may influence the potential supply of reviewers. We estimated the potential supply of reviews by using the relation $N_{reviews-supply}=N_{reviewers-supply}\times \sum_{j=1}^{J}P_{j}\times j$.

We obtained $\sum_{i=1}^{I}R_{i}$ and the empirical distribution of the time taken to perform each review from the 2009 Peer Review Survey, an international survey of 4,037 researchers [8]. Data corresponded to the biomedical domain. We considered *r*_*s*_ to be equal to 2.5 reviewers per peer-review round [11]. We obtained the empirical distribution of individual contributions to the peer-review effort ($\sum_{j=1}^{J}P_{j}$) for 2015 from the Publons reviewer recognition platform. In Publons, reviewers mainly self-report the reviews they have completed (ie, by forwarding review receipts to them). Publons was launched in 2012 and thus we could not obtain data for all unique years of our analysis. We assumed that the distribution for 2015 was identical for every year from 1990 to 2015.

To our best knowledge, reliable data pertaining to *β*, *N*_*u*_ and *d* do not exist. We assumed that 90% of the peer-reviewed submissions went through a second round of peer review (*β* = 0.9), the percentage of the finally unpublished papers was equal to the 20% of the total submissions (*N*_*u*_ = *γT*_*s*_, *γ* = 0.20) and that the average proportion of papers desk-rejected was 25% (*d* = 0.25). Table A in S1 Appendix presents the values of the previously mentioned parameters.

For each researcher, we estimated the total amount of time available for research *t*_*res*_, taking into account whether the researcher was full or part time. We used empirical data provided by the National Institute of Health and Medical Research of France (INSERM), which pertains to all its researchers. The total time spent in peer review was estimated by sampling the respective empirical distribution over the amount of reviews (*j*) completed by each reviewer. For example, if 65% of reviews required 1 to 5 hours to complete, 22% of them 6 to 10 etc., then for each review that a reviewer performed we first drew at random the duration range: between 1 and 5 hours with probability 65%, between 6 and 10 with probability 22%, etc. Afterwards, the actual review time was drawn from a uniform distribution over the interval. Comparing the time devoted to peer review with the total time available for research, we derived the proportion of researchers who devoted certain proportions of their time to peer review (full time, 50% or 30% of their annual work-time). For full-time workers, we used *work time* = 8 *hours/day*, *year* = 365 *days* and *weekends* = 104 *days*. We derived the amount of holidays by averaging between 21 OECD countries (*holidays* = 25.3 *days*) [15]. For each full-time employed researcher, we obtained *t*_*res*_ = 1,885 *hours* and for part-time researchers *t*_*res*_ = 943 *hours* and *t*_*res*_ = 566 *hours* for those devoting 50% and 30% of their time to research, respectively.

### Sensitivity analyses

We performed 25 sensitivity analyses in addition to our main analysis (Table A in S2 Appendix). We used distributions of peer-review effort other than Publons 2015. Under the same conditions, we obtained the respective distributions from Publons for the years 2013 and 2014. We also used a review effort distribution from only a single journal (Nature Materials 2002–2012). Publons data concerned in total about 70,000 researchers and more than 10,000 journals, whereas data from Nature Materials concerned about 4,500 researchers and a single journal. Finally, we varied the values of the parameters (β, γ, d). We summarized the results of all sensitivity analyses by using the relative difference between the annual number of potential reviewers and the annual demand.

## Results

### Main analyses

**Fig 2:**
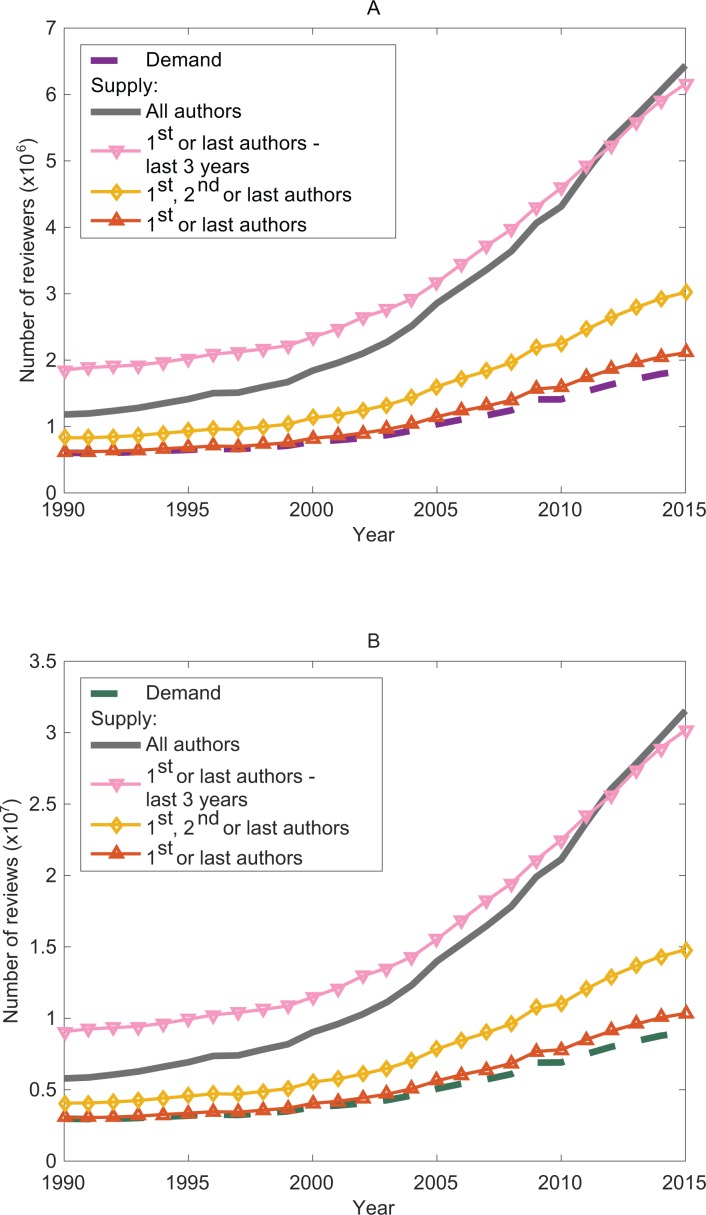
Comparison between supply and demand for reviewers and reviews. (A) Supply and demand for reviewers for all author scenarios. (B) Supply and demand for reviews for all author scenarios.

From 1990 to 2015, the demand for reviews and reviewers was always lower than the supply (Fig 2). In 2015, 1.1 million journal articles were indexed by MEDLINE and we estimated that they required about 9.0 million reviews and 1.8 million reviewers. In contrast, depending on the scenario, the annual supply would be between 10 and 30 million reviews and between 2.1 and 6.4 million reviewers. A substantial proportion of researchers do not contribute to the peer-review effort. In fact, the supply exceeded the demand by 249%, 234%, 64% and 15%, depending on the scenario. The peer-review system in its current state seems to absorb the peer-review demand and be sustainable in terms of volume.

**Fig 3:**
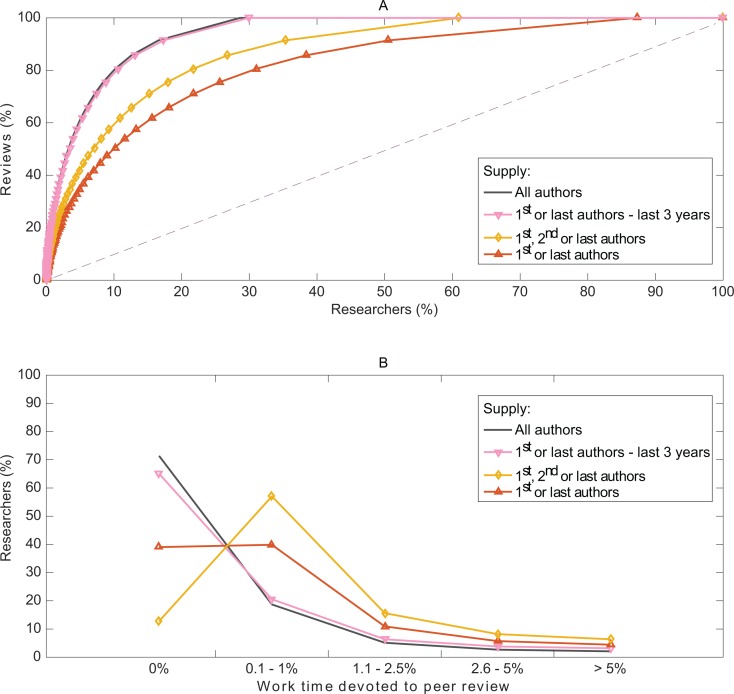
Imbalance in the peer-review effort in terms of workload and work-time. (A) Percentage of authors who complete a certain proportion of the peer-review workload for 2015. (B) Authors’ annual percentage of work-time devoted to peer review.

If the peer-review effort were split equally among researchers, it would generate a demand for 1.4 to 4.2 yearly reviews per researcher, depending on the scenario. However, we found a considerable imbalance in the peer-review effort in that 20% of researchers perform 69% to 94% of reviews (Fig 3A). The imbalance translates into the time spent on peer review. In all, 70% to 90% of researchers dedicate 1% or less of their research work-time to peer review (Fig 3B). Among researchers actually contributing to peer review, 5% dedicate 13% or more of their research work-time to peer review. In 2015, we estimated that a total of 63.4 million hours were devoted to peer review, among which 18.9 (30%) million hours were provided by the top 5% contributing reviewers.

### Sensitivity analyses

When using data from Publons 2014 and 2013, all scenarios to define potential reviewers produced a peer-review supply greater than the demand, except under the most stringent scenario (first or last authors for the same year), in which the demand was higher than the supply before 2001 and 2011, respectively (Fig A in S2 Appendix). For 2015 the supply exceeded the demand by 30% and 35%, respectively, when accounting for first, second or last authors and by 0.5% and 5% when accounting for only first or last authors.

When using data from Nature Materials, the scenarios in which all co-authors for the same year and first or last authors for the last 3 years produced a peer-review supply greater than the demand (the second after 1999). As compared with the most stringent scenarios (first, second or last authors and first or last authors for the same year), these data produced a peer-review demand greater than the supply (Fig A in S2 Appendix). For 2015, the supply exceeded the demand by about 30% for both less-stringent scenarios and the demand exceeded the supply by 120% for the most-stringent scenario. However, this is an extreme distribution covering only a single journal.

When varying the values of γ, the peer-review supply was greater than the demand for all scenarios, except for some values > 0.20 when using the most stringent scenario (Figs C and D in S2 Appendix). Variations over the values of β and d also produced a greater supply than demand for all scenarios (except for d = 0.20 before 2000) (Figs B and E in S2 Appendix). Almost all sensitivity analyses for the last 3 years produced a surplus in number of available reviewers, even though we compared them to the smallest pool of potential peer reviewers (apart from the one of Nature Materials for the two most stringent scenarios, and for two values of γ in the most stringent scenario). Those that produced a deficit as compared to the most stringent scenario, always produced a surplus as compared to the immediately less-stringent one.

## Discussion

Our results challenge recent claims that the growth in published articles may overburden the capacity of the scientific community to absorb the required peer reviews. For the first time, we provide an estimated range for the overall quantitative demand and supply in peer review. The estimation of the quantitative supply we provide refers to the maximum number of reviewers who can be reached by editors according to scenarios and without accounting for individual interactions between authors, editors and reviewers. The scientific community may in fact be able to collectively meet a much higher demand for peer review. This finding is in line with the conclusions of the report of House of Commons Science and Technology Committee and with previous studies in specific journals which showed that peer review was not in crisis [16–19]. However, we showed that a small portion of the scientific community is carrying a disproportionate load of the peer review. These findings are reminiscent of the Pareto principle– 80% of the effects come from 20% of the causes–given that a small number of researchers handles almost all peer reviews. This inequality may be the root of a potentially unmanageable burden. These “peer-review heroes” may be overworked, with risk of downgraded peer-review standards [20].

The geographical distribution of researchers and contributors to the peer-review effort probably explains part of the inequality. In fact, data from two major publishers, Elsevier and Wiley, suggest that, for instance, the proportion of global reviews performed by US researchers is larger than the proportion of global articles they publish. Conversely, Chinese researchers seem to publish twice as many articles as the number they are peer reviewing, despite their willingness to peer review [12, 21].

Peer review should be a collective effort. Reviewing of scientific manuscripts is usually seen as a voluntary and ethical contribution to science, working on a quid pro quo basis. Various reward and incentive systems have been proposed to bolster a more balanced participation in peer-review activities [22, 23]. Reviewer recognition platforms (such as Publons or the Reviewer Recognition Platform) have been launched recently to track and credit peer reviews [24]. Some have suggested offering cash rewards to reviewers or discounts on article processing charges for their future submissions [25]. Such incentives may actually change reviewer motivations and behaviors. Instead, the criteria by which researchers are rewarded for peer-review may be congruent with the more general PQRST system to appraise and reward research, with high-quality transparent peer reviews [26, 27]. Besides, some researchers may be willing to contribute but are never invited. An automated method to improve the matching between submitted articles and the most appropriate candidate peer reviewers may be valuable to the scientific publication system. Such a system could track the number of reviews performed by each author to avoid overburdening them.

Alternative systems of peer review proposed to improve the peer-review system and reduce the burden include “cascade” or “portable” peer review, which would forward the reviews to subsequent journals when papers are resubmitted after being rejected, thus reducing the number of required reviews [28]. Others have suggested re-review opt-out editorial policy or immediate publication with post-publication peer review [29]. A factor that further burdens the peer-review system is the practice of "journal shopping", whereby researchers first target journals with high impact factor and, after rejection, resubmit to journals with gradually lower impact factors. Some initiatives aimed at decreasing journal shopping may contribute significantly to decreasing the overall number of submissions and thus the editorial and peer-review process and the reformatting of manuscripts [30–32].

Here, we focused on journal peer review, but other forms of peer review are likely to impose additional workload on researchers. In particular, the grant peer review system has also been suggested to place a high burden on reviewers. Grant applications may require more work than manuscripts and come in collections at a time because of fixed milestones for submission and deadline systems. Whether the criticism is valid is unclear because empirical evidence concerning the burden on individual researchers and reviewers over time is also scarce [33]. Modeling has been used to address such questions in the grant peer-review system. A recent modeling study from the Office of Extramural Research at the US National Health Institutes (NIH) suggested that the NIH has not tapped the full capacity of the peer-review system [34]. Bollen and colleagues proposed a distributed system and, based on agent-based simulations, showed that the proposed system would result in a similar funding distribution but in less time and cost than the current peer-review system [35, 36].

Our analysis has limitations. First, we assessed the overall quantitative demand and supply and we could not address the qualitative demand and supply. Reviewers are invited by editors on the basis of their expertise in the relevant research area and methodology. “Good” reviewers are likely more solicited for peer review. This situation may explain why the peer-review burden is concentrated on a small portion of researchers. In a survey in political science, 8% of researchers declined requests to review because they considered that they were not sufficiently expert [37]. Moreover, in assessing the supply for peer review, we did not consider that collaborators or scholars from the same institution, for example, may not review each others’ papers or that editors who are also co-authors may not perform additional peer review; these individual interactions between authors, editors and reviewers may reduce to some extent the number of potential reviewers. Instead, we explored this possibility by varying the definition of the pool of potential reviewers according to the ranks of co-authors. Finally, we have not modeled the peer review system as a competitive market economy. In particular, we did not consider the price for peer review and how market forces would apply [38].

Second, we focused on the biomedical literature and our results may not apply to other domains. Even though each discipline has its own characteristics, the biomedical domain accounts for about 44% of the global scientific publications in 2015, and our findings may have implications for domains beyond biomedical research.

Third, we acknowledge that the reliability of our results depends on the data used to inform the modeling. Publons data may not be representative of the true distribution of the peer review effort; registered researchers, who self-report their reviews, may be more intrinsically motivated and more likely to do more reviews than unregistered researchers. To our best knowledge, Publons is the only large-scale source of data about the peer review effort, with data for more than 70,000 reviewers and more than 10,000 journals. We have no data to exclude confidently any selection bias in registered Publons researchers, if any; however, the distribution in Fig 1B shows that 42% of reviewers have reported a single review in 2015. Moreover, Publons has partnered so far with 13 publishers (including Wiley and SAGE), for which registered users automatically receive credit for the reviews they performed (86,910 reviews from 2,676 journals). This finding goes against an overrepresentation of more active reviewers.

We have conducted sensitivity analyses based on data from one specific journal (Nature Materials)[39]. We observed surplus potential reviewer supply when all authors and when the first or last authors across the last 3 years were eligible as reviewers; under more stringent assumptions (1st, 2nd or last and 1st or last author within 1 year), we found a deficit in the reviewer supply. However, researchers are likely to be invited and review for more than one journal; as a consequence, that distribution probably underestimates the effort distribution.

As well, we have used data from the Peer Review Survey to inform the distribution of resubmissions before publication. Although these are the only data about the whole resubmission pattern, they are also limited by self-reporting and a response rate of about 10%. Calcagno et al. previously documented the late submission history of 80,748 articles in biological sciences (self-reporting data with a response rate of 37%) and found that about 75% of published articles were submitted first to the journal that published them.[30].

Another limitation is that our analysis relied on assumptions. However, we restricted these assumptions only to cases when empirical data were, to our best knowledge, not available. In such cases, we set arbitrary but pre-specified values and the values were chosen to reflect realistic scenarios; we performed sensitivity analyses, extensively exploring the parameter space and obtaining results mostly similar to our main analysis (as shown in the S2 Appendix).

One might be interested in analyzing sub-communities of the biomedical system, such as reports of clinical trials. Our search of MEDLINE could have been easily restricted to a smaller selection of articles to reflect these sub-communities. However, summing up the results of all specific sub-communities would give similar results as those obtained from analyzing the whole biomedical domain. Finally, our analysis is also limited by potential issues in the indexation of author names in MEDLINE. Multiple individual researchers can share the same “LastName”, “ForeName”, “Initials” triplet. Conversely, a given individual researcher could appear as several researchers because of misspellings. We acknowledge that we did not use algorithmic author name disambiguation [13]. The first type of error would lead to underestimating the number of potential reviewers and the second to overestimating the number of potential reviewers. These two types of errors are antagonistic–their effects could be cancelled out–but their impact on our results is difficult to quantify.

In conclusion, the current peer-review system is sustainable in terms of volume but the distribution of the peer-review effort is substantially imbalanced across the scientific community. The evidence base for alternative peer-review systems is still sparse [40, 41]. An evidence-based approach to study peer review, combining computer modeling, experimental studies and sharing of data from journals and publishers, should be encouraged [42–45]. Improvements in peer review will come in response to evidence.[46]

## Supporting Information

S1 Appendix Analytical methods. (PDF)

S2 Appendix Sensitivity analyses. (PDF)
