# Supplementary material for: The Global Burden of Journal Peer Review in the Biomedical Literature: Strong Imbalance in the Collective Enterprise
Source: PLoS One. 2016 Nov 10;11(11):e0166387. doi: 10.1371/journal.pone.0166387 (PMC5104353; doi:10.1371/journal.pone.0166387)
Supplement: S1 Appendix — (PDF) [file pone.0166387.s001.pdf]

S1 Appendix. Analytical methods

In this section, we first present how we estimated the number of submissions, the demand for peer review, the supply of peer review and the time researchers devote to peer review. Then we present the data we used to inform our modelling. Finally, we present sensitivity analyses over different distributions and values.

Estimation of demand and supply for peer-review

Let us consider N_p the number of articles accepted for publication. Let N_u be the number of articles submitted for publication but that ultimately remain unpublished. We accounted for multiple submissions after rejections, which all occurred within a given year. We assumed that both published and unpublished papers followed the same distribution of resubmissions. Let us define $R_i{}'$, the proportion of manuscripts submitted exactly *i* times. The proportion of manuscripts submitted at least i times is $R_i = \sum_{k \geq i} R'_{k-1}$. Then the total number of submissions is:

$$N_s = \left(N_p + N_u\right) \times \sum_{i=1}^{I} R_i \times i \qquad (1)$$

For simplicity, we set a maximum amount of resubmissions (*I*). For example, if 5% of papers are submitted once, 10% are submitted twice and 85% are submitted three times, then $R'_1 = 0.05$, $R'_2 = 0.10$, $R'_3 = 0.85$, $R_1 = 1$, $R_2 = 0.95$, and $R_3 = 0.85$. Then, $\sum_{\boldsymbol{i}=\mathbf{1}}^{\mathbf{3}} \boldsymbol{R_i} \times \boldsymbol{i} = 1 \times 1 + 0.95 \times 2 + 0.85 \times 3 = 5.45$. If we further assume that 800 manuscripts were ultimately published and 200 ultimately unpublished, the total number of submissions is $N_s = 800 \times (1 + 0.95 \times 2 + 0.85 \times 3) + 200 \times (1 + 0.95 \times 2 + 0.85 \times 3) = 1{,}000 \times 5.45 = 5{,}450\ submissions$.

The distribution of resubmissions of published and unpublished papers might differ, but we can transform it to be the same:

$$N_u{}^0 \times \sum_{i=1}^{I} R_i^0 \times i = N_u{}^0 \times \alpha \times \sum_{i=1}^{I} R_i \times i = N_u \times \sum_{i=1}^{I} R_i \times i \qquad (2)$$

where α is a constant, $\mathrm{N_u}{}^0 = \frac{N_u}{\alpha}$ the real amount of unpublished papers and $\mathrm{R_i^0}$ the real proportion of papers (re)submitted i times but never published. For example, if $\mathrm{R_1^0} = 1$, $\mathrm{R_2^0} = 0.85$, and $\mathrm{R_3^0} = 0.55$, then $\sum_{\boldsymbol{i}=\mathbf{1}}^{\mathbf{3}} \mathrm{R_i^0} \times \boldsymbol{i} = 4.35$. If $\mathrm{N_u}{}^0 = 100$, then the total number of submissions which did not result in a publication is 370. In reality we do not know both $\sum_{\mathrm{i}=1}^{\mathrm{I}} \mathrm{R_i^0} \times \mathrm{i}$ and $\mathrm{N_u}{}^0$ and it would be impossible to obtain reliable data for them. However, we know $\sum_{\mathrm{i}=1}^{\mathrm{I}} \mathrm{R_i} \times \mathrm{i}$ and we can represent $\sum_{\mathrm{i}=1}^{\mathrm{I}} \mathrm{R_i^0} \times \mathrm{i}$ in terms of it using a constant α. Then, we can group α and $\mathrm{N_u}{}^0$ into a single constant $\mathrm{N_u}$ and work with equation 1.

We estimated the annual demand for reviews $\mathrm{N_{reviews}}$ as:

$$N_{reviews} = (1 - d) \times r_s \times (N_s + \sum_{i=1}^{I} S_i) \qquad (3)$$

where d is the proportion of desk-rejected submissions, r_s the number of reviewers per peer review round and S_i the amount of papers that went to a second round of peer review in their i^{th} (re)submission. We defined S_i as follows:

$$S_i = \beta \times (N_p + N_u) \times R_i \qquad (4)$$

where β is the probability of a second peer-review round per submission that is not desk-rejected.

We can estimate $N_{reviews}$ using a different formula, which this time involves the annual demand for reviewers $N_{reviewers}$.

$$N_{reviews} = N_{reviewers} \times \sum_{j=1}^{J} P_j \times j \qquad (5)$$

where J is the maximum amount of annual reviews that any reviewer performed, j the amount of reviews completed from a reviewer in a given year and P_j the proportion of reviewers who completed j reviews. For example, if 1,000 scientists reviewed at least one paper inside a year, 60% of them performed 1 and 40% of them 2 reviews, then $\mathrm{N_{reviews}} = 1000 \times (0.6 \times 1 + 0.4 \times 2) = 1{,}400\ reviews$. Since we have two formulas estimating $N_{reviews}$, we can estimate the annual demand for reviewers from their combination:

$$N_{reviewers} = \frac{N_{reviews}}{\sum_{j=1}^{J} P_j \times j} = \frac{(1-d) \times r_s \times (N_s + \sum_{i=1}^{I} S_i)}{\sum_{j=1}^{J} P_j \times j} \qquad (6)$$

We defined each researcher's total amount of time available for research as follows:

$$t_{res} = work\ time \times (year - weekends - holidays) \qquad (7)$$

Collection and use of data

All data and results can be found in the accompanying Excel file (http://www.clinicalepidemio.fr/peerreview_burden/). We programmed our simulations by using MATLAB (MATLAB and Statistics Toolbox Release 2014b, The MathWorks, Inc., Natick, MA, USA). The code is available at https://github.com/kovanostra/global-burden-of-peer-review.

We used data pertaining to the biomedical domain, except to estimate r_s and the distribution of peer-review effort ($\sum_{j=1}^{J} P_j$), for which we used data pertaining to all scientific disciplines. We extracted all records indexed as "journal articles" by MEDLINE from January 1, 1990 to December 31, 2015. We downloaded the xml files for each year separately and parsed them by using a script written in Python (also available on github). We excluded all records with no author name (*e.g.,* less than 0.001% of all articles for 2015) and indexed all authors based on their

"LastName", "ForeName" and "Initials". We counted all the unique occurrences of authors by taking into account all these three pieces of information. For missing "ForeName" and/or "Initials", we used only the available fields. We did not use any methods for author name disambiguation for researchers indexed under the same "LastName", "ForeName" and "Initials".[1, 2] We set N_s to be equal to the number of publications for which we identified at least one author.

We assumed that potential reviewers in a given year were researchers who co-authored at least one paper that year (Scenario 1). Then we defined more stringent scenarios (in terms of which co-authors are potential reviewers) whereby candidate reviewers were the first or last authors of any article during the previous 3 years (Scenario 2); the first, second or last authors for the same year (Scenario 3); and the first or last authors for the same year (Scenario 4). For Scenario 2, we arbitrarily chose a time window of 3 years, which however may reflect changes in the databases that editors use to find reviewers. For each scenario, we repeated the same procedure of identifying the unique occurrences of authors as described above. For each scenario, the number of authors obtained was considered to represent the potential supply of reviewers ($N_{reviewers-supply}$) in any given year. We did not account for individual interactions between authors, editors and reviewers which may influence the potential supply of reviewers. We estimated the potential supply of reviews by using the relation $N_{reviews-supply} = N_{reviewers-supply} \times \sum_{j=1}^{J} P_j \times j$.

We obtained $\sum_{i=1}^{I} R_i$ and the empirical distribution of the time taken to perform each review from the 2009 Peer Review Survey, an international survey of 4,037 researchers [3]. Data corresponded to the biomedical domain. We considered r_s to be equal to 2.5 reviewers per peer-review round [4]. We obtained the empirical distribution of individual contributions to the peer-review effort ($\sum_{j=1}^{J} P_j$) for 2015 from the Publons reviewer recognition platform. In Publons, reviewers mainly self-report the reviews they have completed (ie, by forwarding review receipts to them). Publons was launched in 2012 and thus we could not obtain data for all unique years of our analysis. We assumed that the distribution for 2015 was identical for every year from 1990 to 2015. To our best knowledge, reliable data pertaining to β, N_u and d do not exist. We assumed that 90% of the peer-reviewed submissions went through a second round of peer review ($\beta = 0.9$), the percentage of the finally unpublished papers was equal to the 20% of the total submissions ($N_u = \gamma T_S, \gamma = 0.20$) and that the average proportion of papers desk-rejected was 25% ($d = 0.25$). Table A presents the values of the previously mentioned parameters.

Table A: Parameter values

Variable	Description	Value	Source

r_s	Average reviewers per paper	2.5	Reference 4
β	Chance of second peer-review round	90%	No reference – Sensitivity analyses performed
γ	Proportion of unpublished papers among all submissions	20%	No reference – Sensitivity analyses performed
d	Average proportion of desk-rejected papers	25%	No reference – Sensitivity analyses performed
holidays	Holidays	25.3	Reference 5

For each researcher, we estimated the total amount of time available for research t_{res}, taking into account whether the researcher was full or part time. We used empirical data provided by the National Institute of Health and Medical Research of France (INSERM), which pertains to all its researchers. The total time spent in peer review was estimated by sampling the respective empirical distribution over the amount of reviews (j) completed by each reviewer. For example, if 65% of reviews required 1 to 5 hours to complete, 22% of them 6 to 10 etc., then for each review that a reviewer performed we first drew at random the duration range: between 1 and 5 hours with probability 65%, between 6 and 10 with probability 22%, etc. Afterwards, the actual review time was drawn from a uniform distribution over the interval. Comparing the time devoted to peer review with the total time available for research, we derived the proportion of researchers who devoted certain proportions of their time to peer review (full time, 50% or 30% of their annual work-time). For full-time workers, we used $work\ time = 8\ hours/day$, $year = 365\ days$ and $weekends = 104\ days$. We derived the amount of holidays by averaging between 21 OECD countries ($holidays = 25.3\ days$) [5]. For each full-time employed researcher, we obtained $t_{res} = 1{,}885\ hours$ and for part-time researchers $t_{res} = 943\ hours$ and $t_{res} = 566\ hours$ for those devoting 50% and 30% of their time to research, respectively.

References

1. Schulz C, Mazloumian A, Petersen AM, Penner O, Helbing D. Exploiting citation networks for large-scale author name disambiguation. EPJ Data Science. 2014;3(1):1-14. doi: 10.1140/epjds/s13688-014-0011-3.
2. Lerchenmueller MJ, Sorenson O. Author Disambiguation in PubMed: Evidence on the Precision and Recall of Author-ity among NIH-Funded Scientists. PLoS ONE. 2016;11(7):e0158731.

3. Mulligan A, Hall L, Raphael E. Peer review in a changing world: An international study measuring the attitudes of researchers. J Assoc Inf Sci Technol. 2013;64(1):132-61. doi: 10.1002/asi.22798.
4. Look H, Sparks S. The value of UK HEIs contribution to the publishing process: Summary report: Rightscom Ltd for JISC Collections; 2010. Available from: http://www.jisc-collections.ac.uk/Reports/valueofukhe/.
5. Ray R, Schmitt J. No-vacation nation USA – a comparison of leave and holiday in OECD countries. EEE Policy Brief. 2007;3.
