# Supplementary material for: The Global Burden of Journal Peer Review in the Biomedical Literature: Strong Imbalance in the Collective Enterprise
Source: PLoS One. 2016 Nov 10;11(11):e0166387. doi: 10.1371/journal.pone.0166387 (PMC5104353; doi:10.1371/journal.pone.0166387)
Supplement: S2 Appendix — (PDF) [file pone.0166387.s002.pdf]

S2 Appendix. Sensitivity analyses

We performed 25 sensitivity analyses in addition to our main analysis (Table A). In the first 3 analyses, we used distributions of peer-review effort other than Publons 2015. Under the same conditions, we obtained the respective distributions from Publons for the years 2013 and 2014, corresponding again to all scientific domains. We also used a review effort distribution from only a single journal (*Nature Materials* 2002-2012).[1] Publons data concerned in total about 70,000 researchers and more than 10,000 journals, while data from Nature materials concerned about 4,500 and a single journal. For the remaining 22 sensitivity analyses, we varied the values of the parameters (*β, γ, d*) while using only the distribution from Publons 2015.

We evaluated all our sensitivity analyses under one outcome, the surplus in the annual number of potential reviewers as compared with the annual demand. For sensitivity analysis 1, we explored the possible surplus in the number of potential reviewers for each of the four scenarios as compared to the respective demand. For sensitivity analyses 2 to 25, we defined the surplus by using only scenario 4 and in some cases scenario 3 as well.

The distribution from *Nature Materials*, when using scenarios 1 and 2, produced a surplus for any given year (scenario 2 after 1999). However, it produced a deficit when considering scenarios 3 and 4 for any given year (Fig A). When using scenario 4 the distributions of the peer-review effort from Publons for 2014 and 2013 produced surplus in the potential supply of reviews and reviewers when compared to scenario 3, for any given year, and when compared to scenario they produced surplus after 2001 and 2011, respectively, (Fig A).

For most of the values of *γ*, we found a surplus in the number of available reviewers as compared to scenario 4, and for all of them when compared to scenario 3 (Figs C and D). Variations over the values of *β* and *d* did not produce any deficit when compared to scenario 4 (except for d = 0.20 and before 2000) (Figs B and E). Almost all sensitivity analyses (apart from the one of Nature Materials) for the last 3 years produced a surplus in the number of available reviewers, even though we compared them to the smallest pool of potential peer reviewers. Those that produced deficit when compared to scenario 4, always produced surplus when compared to scenario 3.

Table A: Sensitivity analyses

**Sensitivity analysis**	**Peer-review effort distribution**	**Desk rejection proportion *(d)***	**Proportion of unpublished submissions to all submissions *(γ)***	**Probability of second round of reviews *(β)***
**Main analysis**	Publons 2015	0.25	0.200	0.90
**1**	Nature Materials	″	″	″
**2**	Publons 2013	″	″	″
**3**	Publons 2014	″	″	″
**4**	Publons 2015	0.20	″	″
**5**	″	0.30	″	″
**6**	″	0.35	″	″
**7**	″	0.40	″	″
**8**	″	0.45	″	″
**9**	″	0.50	″	″
**10**	″	0.55	″	″
**11**	″	0.60	″	″
**12**	″	0.25	0.100	″
**13**	″	″	0.135	″
**14**	″	″	0.170	″
**15**	″	″	0.235	″
**16**	″	″	0.270	″
**17**	″	″	0.300	″
**18**	″	″	0.335	″
**19**	″	″	0.370	″
**20**	″	″	0.200	0.60
**21**	″	″	″	0.65
**22**	″	″	″	0.70
**23**	″	″	″	0.75
**24**	″	″	″	0.80
**25**	″	″	″	0.85

Distribution of peer-review effort and values for *d, γ, β* from the main analysis and from all sensitivity analyses.

Sensitivity analyses 1–3

Figure A. Sensitivity analyses by distributions of peer-review effort

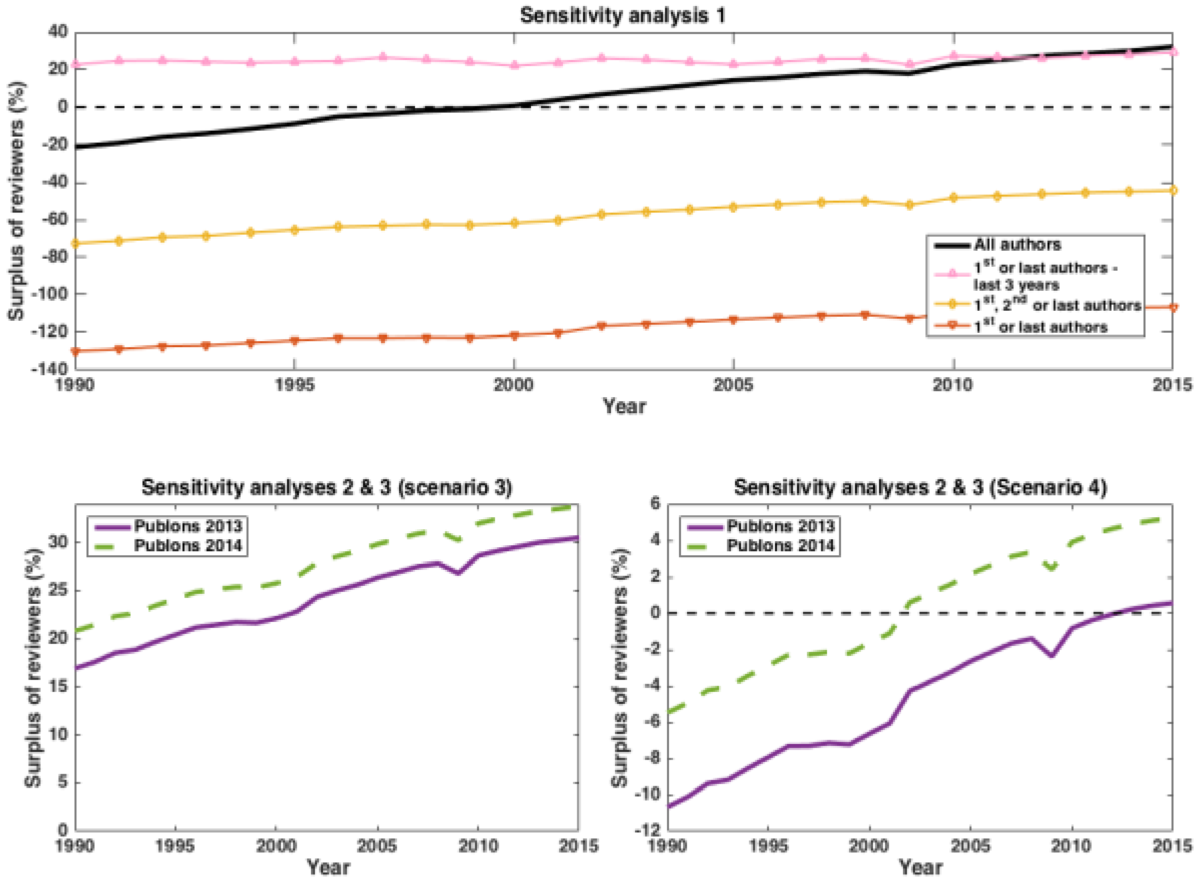

A. Sensitivity analysis involved the distribution from *Nature Materials* (2002–2012). Surplus defined with all scenarios 1–4 to identify the potential supply of reviewers. **B.** Sensitivity analyses involved the distributions from Publons for the years 2013 and 2014. Surplus defined with only scenarios 3 and 4 to identify the potential supply of reviewers.

Sensitivity analyses 4–11

Figure B. Sensitivity analyses by desk-rejection rate

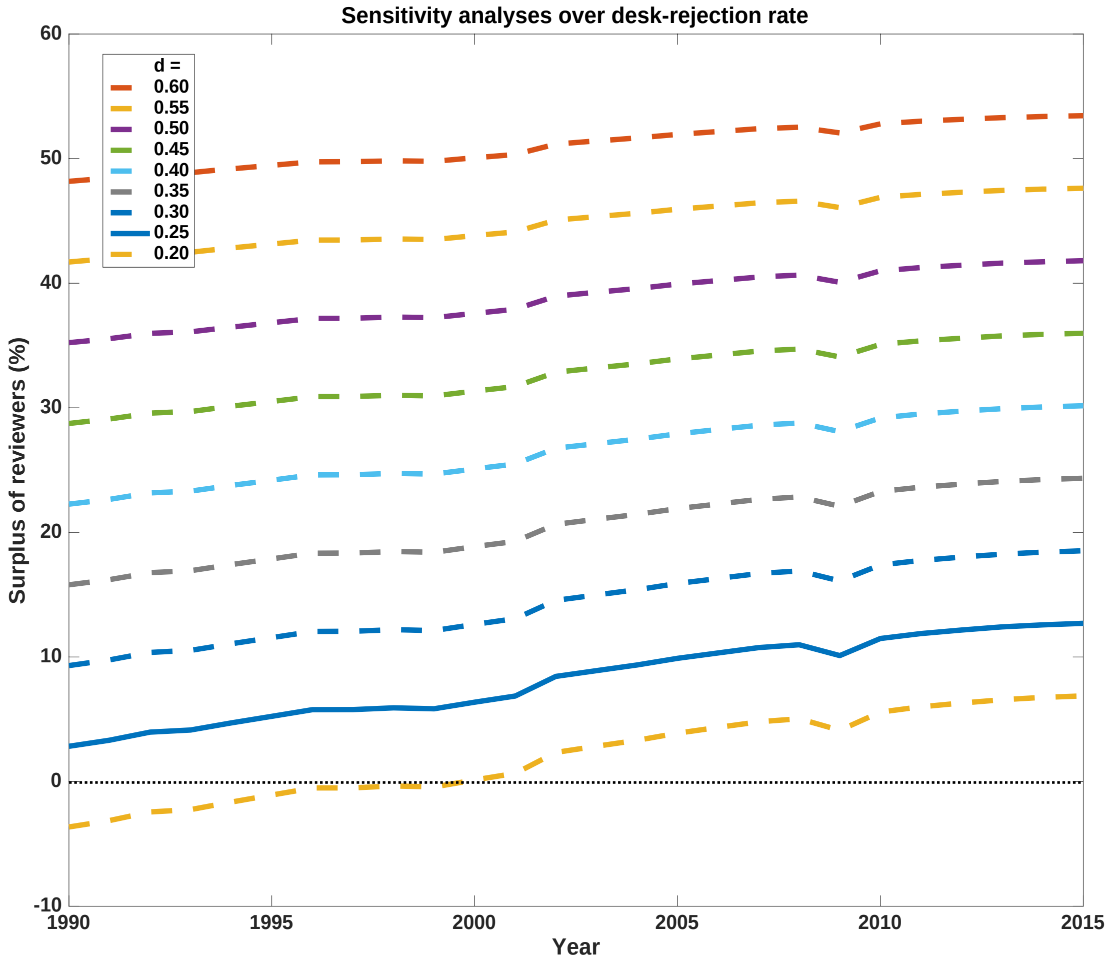

Sensitivity analyses of different values of the overall proportion of desk-rejected manuscripts per submission. The continuous line shows the value used in the main analysis.

Sensitivity analyses 12–19

Figure C. Sensitivity analyses by proportion of unpublished papers (Scenario 3)

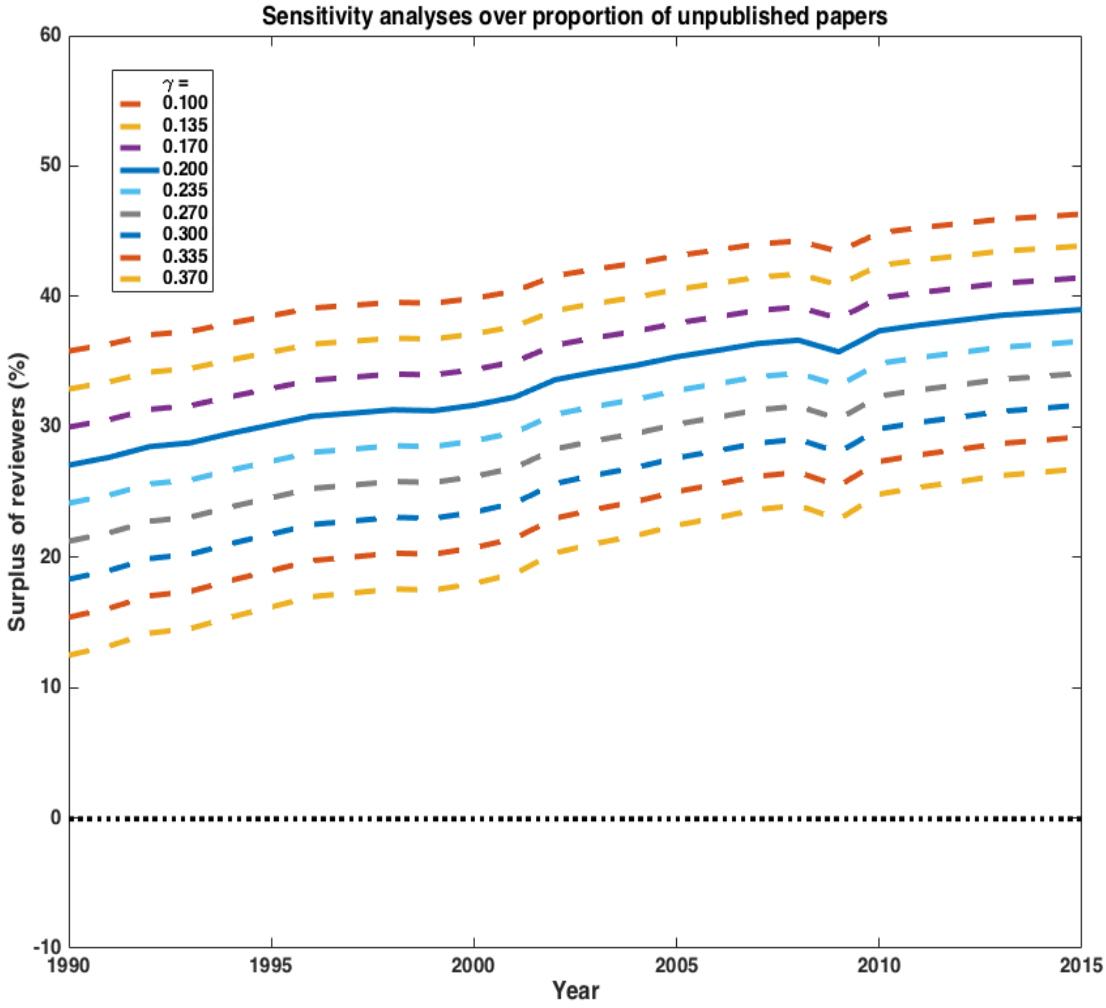

Sensitivity analyses of different values of the proportion of unpublished papers compared to overall submissions. Surplus defined with scenario 3 to identify the potential supply of reviewers. The continuous line shows the value used in the main analysis.

Sensitivity analyses 12–19

Figure D. Sensitivity analyses by proportion of unpublished papers (Scenario 4)

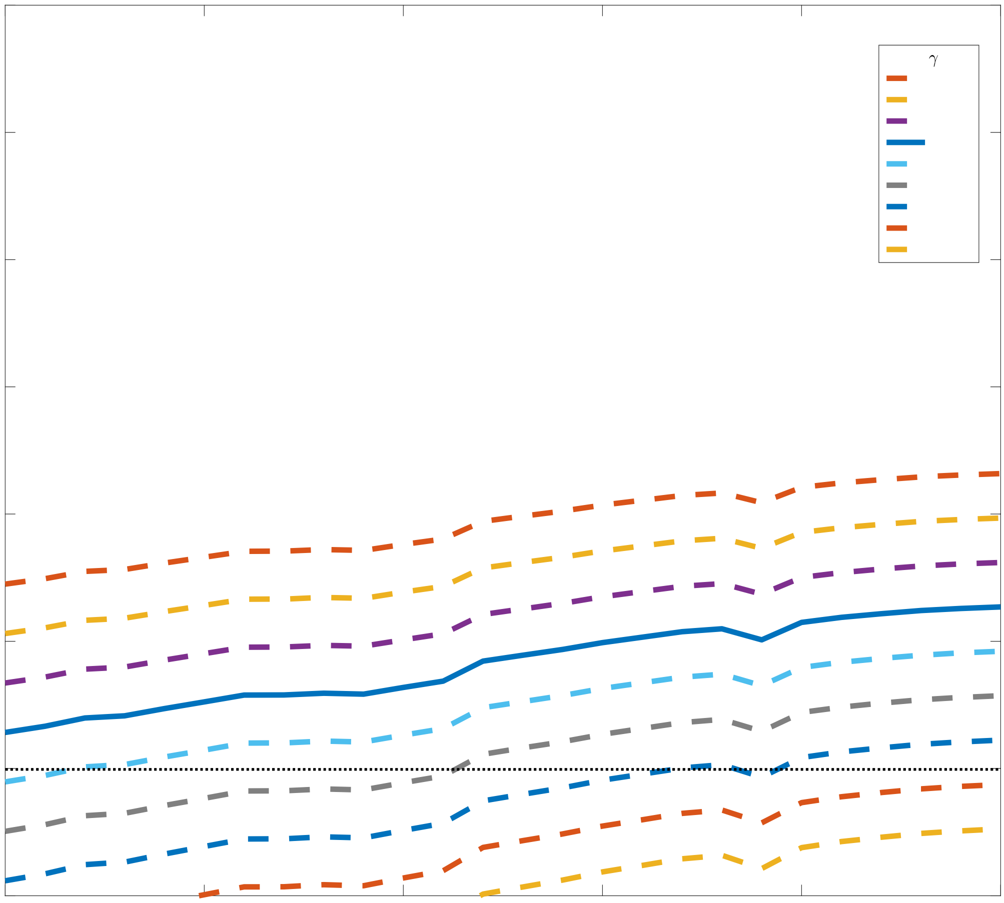

Sensitivity analyses of different values of the proportion of unpublished papers compared to overall submissions. The continuous line shows the value used in the main analysis.

Sensitivity analyses 20–25

Figure E. Sensitivity analyses by probability of second round of reviews

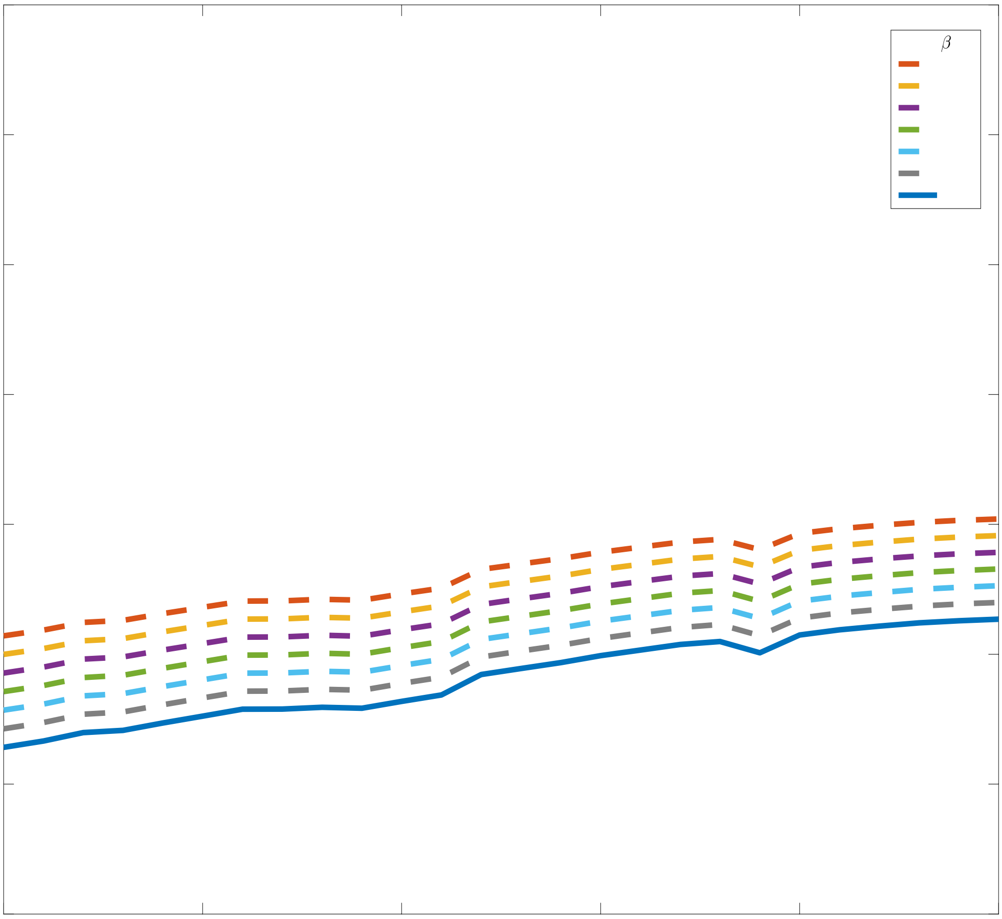

Sensitivity analyses of different values of the probability of papers going through a second round of peer review for a submission that was not desk-rejected. The continuous line shows the value used in the main analysis.

References

1. A decade in numbers. Nat Mater. 2012;11(9):743-4.
